# Medical schools in India: pattern of establishment and impact on public health - a Geographic Information System (GIS) based exploratory study

**DOI:** 10.1186/s12889-020-08797-0

**Published:** 2020-05-24

**Authors:** Yogesh Sabde, Vishal Diwan, Vijay K. Mahadik, Vivek Parashar, Himanshu Negandhi, Tanwi Trushna, Sanjay Zodpey

**Affiliations:** 1 National Institute for Research in Environmental Health, Bhopal, Madhya Pradesh India; 2 Department of Global Public Health, Karolinska Institutet, Stockholm, Sweden; 3 R.D.Gardi Medical College Ujjain, Ujjain, Madhya Pradesh India; 4 Indian Institute of Public Health, New Delhi, India; 5 Public health Foundation of India, New Delhi, India

**Keywords:** National Medical Commission bill, Medical school, Geographic information system, Near neighbourhood analysis, Distance matrix, India

## Abstract

**Background:**

Indian medical education system is on the brink of a massive reform. The government of India has recently passed the National Medical Commission Bill (NMC Bill). It seeks to eliminate the existing shortage and maldistribution of health professionals in India. It also encourages establishment of medical schools in underserved areas. Hence this study explores the geographic distribution of medical schools in India to identify such under and over served areas. Special emphasis has been given to the mapping of new medical schools opened in the last decade to identify the ongoing pattern of expansion of medical education sector in India.

**Methods:**

All medical schools retrieved from the online database of Medical Council of India were plotted on the map of India using geographic information system. Their pattern of establishment was identified. Medical school density was calculated to analyse the effect of medical school distribution on health care indicators.

**Results:**

Presence of medical schools had a positive influence on the public health profile. But medical schools were not evenly distributed in the country. The national average medical school density in India amounted to 4.08 per 10 million population. Medical school density of provinces revealed a wide range from 0 (Nagaland, Dadra and Nagar Haveli, Daman and Diu and Lakshadweep) to 72.12 (Puducherry). Medical schools were seen to be clustered in the vicinity of major cities as well as provincial capitals. Distance matrix revealed that the median distance of a new medical school from its nearest old medical school was just 22.81 Km with an IQR of 6.29 to 56.86 Km.

**Conclusions:**

This study revealed the mal-distribution of medical schools in India. The problem is further compounded by selective opening of new medical schools within the catchment area of already established medical schools. Considering that medical schools showed a positive influence on public health, further research is needed to guide formulation of rules for medical school establishment in India.

## Background

Indian medical education system is on the brink of a massive reform. The government of India has recently passed the National Medical Commission Bill (NMC Bill) to create a world class medical education system that ensures adequate supply of high quality of medical professionals in the country while being flexible enough to adapt to the changing needs of a transforming nation [1]. This move is not surprising considering that India has always adhered to the tenet which asserts that the health of the country’s population influences its overall development and its economic prosperity [2]. The country has taken positive steps in strengthening its commitment towards Universal Health Coverage (UHC) proposed by World Health Organization (WHO) [3] by launching the ambitious Ayushman Bharat Health Scheme (Pradhan Mantri Jan Arogya Yojana) in 2018 [4]. However according to the 2017 Global Monitoring Report published by WHO and World Bank, a mal-distributed health workforce prevents achievement of UHC [5]. Health workers who constitute the human resources for health (HRH) are essential for efficient functioning of the health system [6]. WHO thus recommends a minimum of 44.5 health professionals per 10,000 population [7]. Published literature reveals that in India availability of health workers is well below the WHO recommendation [8, 9]. India ranks 52nd among the 57 countries identified by The Global Health Workforce Alliance and WHO which are most severely affected by health workforce crisis [10]. Inadequacies in the size and composition of HRH in India hence has led to inequities in health outcomes [11]. Substantial disparity exists in the health performance of different provinces of the country which closely correlates with the density of health workers available [11].

HRH in India is composed of a varied range of formal and informal healthcare providers of which allopathic doctors form an integral part [12]. According to WHO, at least 10 doctors should cater to 10,000 population [7]. In India the national density of allopathic doctors is 5.9 per 10,000 population of which 24% do not possess adequate training [12]. Scarcity of trained doctors has persisted in the country despite the rapid increase in the number of medical schools making Indian medical education system the largest in the world [13]. National shortage of doctors is juxtaposed with gross mal-distribution resulting in a wide divide in the availability of doctors in urban and rural areas [14]**.**

Boulet et al.(2007) have shown that density of practicing doctors in a region is closely associated with the density of medical schools located within its geographic limits [13]**.** To address the acute deficiency of trained doctors, Government of India has initiated several efforts to expand the medical education sector. Under the Pradhan Mantri Swasthya Suraksha Yojana, 6 new AIIMS (All India Institute of Medical Sciences) have been established in the country, which are now fully functional and 16 more projects have been announced. Seventy five government medical schools have been planned under the same national scheme for development in six phases [15]**.** Five hundred eighty million dollars have been allocated to this scheme for expansion of the Indian medical education system in the 2019–20 financial budget [16]**.**

However as Ananthkrishnan rightly pointed out, merely increasing the number of medical schools in the country and their total annual intake will not solve the health workforce crisis as long as the skewed country-wide distribution of medical schools is not corrected [17]. Previous studies have revealed the uneven distribution of medical schools in India. Mahal et al. in 2006 showed that economically better off provinces of India have higher medical school density [18]. Two subsequent studies have documented the substantial difference in medical school density between southern versus northern and eastern regions of India [17, 19]. Shortage of medical schools in rural districts was documented by Brahmapurkar et al. [20]**.**

The blame for this disparity in medical school distribution in India can be partly attributed to the one-dimensional regulations guiding establishment of new medical schools in India. According to the prevalent MCI (Medical Council of India) norms, the criteria needed to be fulfilled to obtain a no-objection certificate (NOC) from the provincial government for opening a new medical school focused mostly on availability of adequate land and the potential demand rather than on the health needs of the area [21]**.** Hence a comprehensive reform of Indian medical education is the need of the hour. Acknowledging this need, the government of India is readying itself to modernize medical education in the country through promulgation of the National Medical Commission Bill (NMC Bill) [1]. It is proposed in the bill that while permitting establishment of new medical schools the new regulating authority shall have due attention to financial resources, availability of adequate faculty and hospital facilities while also allowing the said criteria to be relaxed for those medical schools which are to be set up in an underserved area. However the bill has not clarified the definition of an underserved area. Therefore it is necessary to clearly demarcate areas which have the lowest medical school density and which would benefit the most by the establishment of a medical school. A search through the literature published on Indian medical education yielded very few studies that have attempted to spatially orient the distribution of medical schools in India [19]. Hence this study explores the geographic distribution of medical schools in India. Special emphasis has been given to the mapping of new medical schools opened in the last decade to identify the ongoing pattern of expansion of medical education sector in India.

Although medical school density has been found to affect regional doctor density, the correlation between the number and distribution of medical schools and population health indicators is not well established especially in the Indian context. As India is poised at the brink of a new era of medical education, it is relevant to explore the association of medical school distribution with the background indicators of the area they serve.

This study was undertaken with the following objectives in mind-

1. To describe the spatio-temporal distribution of all public and private owned medical schools according to their distribution among Indian provinces and their year of inception.
2. To compare the geographic distribution of new medical schools (opened in the last decade i.e. 2009–2019) with that of old medical schools (established before 2009).
3. To compare the socioeconomic and health care profile of districts according to the presence or absence of new and old medical schools.

## Methods

### Study setting

India is a conglomerate of 37 administrative divisions (28 provinces and 9 Union Territories or UTs) [22] with a total population of approximately 1211 million [23]**.** Administrative unit of each of those provinces/UTs is called district. As per to official statistics, India has a total of 640 districts [24]. Each district is composed of varying proportion of urban and rural areas. According to Urban and Regional Development Plans Formulation and Implementation (URDFPI) Guidelines urban areas are further classified into towns and various classes of cities [25]**.**

### Data sources

1. Data on medical schools in India-

This study relied on the online database maintained by the Medical Council of India (MCI) for information regarding the location, year of inception and annual intake of medical schools. Till August 2019 MCI was the regulatory authority that guides medical education in India. Its accreditation was mandatory for establishment and functioning of medical schools [26]**.** The present study included all medical schools in India which provide medical education leading up to the MBBS (medical graduate) qualification, as listed in the official website of MCI as of 22^nd^April 2019 [27].

2. Data on socioeconomic and health profile Indicators-

*Provincial level health indicators*: Province wise values for Infant mortality rate (IMR per 1000 live births) for the year 2016, Institutional deliveries (ID expressed as a percentage of total deliveries) for year 2015–16 and maternal mortality ratio (MMR per 100,000 live births) for the year 2014–16 were retrieved from the official website of National Institution for Transforming India (NITI), Government of India [28–30]. These 3 indicators were specifically chosen keeping in mind the importance of maternal and child health in over all public health of a country and since developing countries like India are concentrating on improving MMR, IMR and ID to help reduce the overall health burden [31].

*District level indicators:* District Fact Sheets of National Family Health Survey - 4 (2015–16, [32]) provide district wise data on many key indicators of reproductive health and family planning, infant and child mortality, maternal and child health, nutrition, anaemia, utilization and quality of health and family planning services. Of all the available indicators we have chosen four representative ones that indicate population and household profile including infrastructure, sanitation, cooking fuel and literacy status of the districts to reflect the socio-economic status of the respective districts. Similarly, data on seven representative health-care indicators was retrieved for use to reflect the public health status of the respective districts. The indicators most likely to be influenced by the access to health care services were used as the effect of presence/absence of medical school will be most evident in these cases. The key indicators chosen have highlighted in Table 1.

**Table 1:** Key socioeconomic and public health indicators chosen for analysis

Domains	Key Indicators	Indicate:
Population and household profile	•Households with electricity, •Households using improved sanitation facility, •Households using clean fuel for cooking, •Women who are literate.	Socio-economic Status of districts
Maternal and child health	•Mothers who had full antenatal care, •Mothers who received postnatal care from a doctor/ nurse/ LHV/ ANM/ midwife/ other health personnel within 2 days of delivery, •Children who received a health check after birth from a doctor/nurse/LHV/ANM/ midwife/other health personnel within 2 days of birth, •Births assisted by a doctor/ nurse/LHV/ANM/other health personnel, •Children with fever or symptoms of ARI in the last 2 weeks preceding the survey taken to a health facility, •Women Age 15–49 Years Who Have Ever Undergone Examinations of Cervix, •Women Age 15–49 Years Who Have Ever Undergone Examinations of Breast.	Public health status of districts

### Mapping

Locations of district headquarters (HQs) and boundaries were available in the digital map of India which was purchased from the office of Survey of India (SOI) in Dehra Dun, India (License No. “BP11CDLA183”). The provincial boundaries of Telangana province were updated from the GADM database (www.gadm.org), version 3.6 (released on 6 May 2018). (Jammu and Kashmir and Ladakh were treated as one administrative division as the boundary maps separating the two and other relevant data were not available in retrieved databases). For analysis, the digital map that we used contained 29 provinces and 7 UTs. Locations of all the medical schools in MCI list were manually digitized by finding their coordinates on base maps of ArcInfo and open access databases like Google Maps and Bing Maps. These data were presented in maps using appropriate symbology.

### Analysis

Using the year of inception as per MCI data, medical schools established before 2009 were termed as ‘old medical schools’ while medical schools opened during 2009 to 2019 were referred to as ‘new medical schools’. Country level distribution of old and new medical schools in public and private sector was plotted as a main map (scale 0 to 500Km). Detailed distribution of geographic clusters of medical schools at major cities and provincial capitals was visualized using insets (scale of 0 to 50 Km) in the same graphic. The ‘count features in a polygone’ algorithm of the QGIS Desktop (version 2.18.24) was used to count the number of old and new medical schools in a geographic territory like Province, UT or district. Near neighbourhood analysis which calculates the Nearest Neighbor ratio (NNR) and its Z score was done to check for statistical significance of the clustering of old as well as new medical schools in India. If the NNR is less than 1 it can be inferred that the pattern exhibits clustering (https://docs.qgis.org/testing/en/docs/user_manual/processing_algs/qgis/vectoranalysis.html#qgisnearestneighbouranalysis). Distance matrix algorithm was used to determine the median distance of a ‘new medical school’ from its nearest ‘old medical school’. (https://docs.qgis.org/2.18/en/docs/training_manual/vector_analysis/spatial_statistics.html?highlight=distance%20matrix).

### Medical school density

Density of medical schools was calculated as the total number of medical schools per 10 million population (as per census 2011). To begin with the national medical school density was calculated. Subsequently, the density of medical schools for each province/UT was also calculated. Choropleth map was used to visualize the province/UT level quintiles of medical school density. Spearman’s rho was used to detect correlation between province/UT level medical school density (Old, New, and Total) and their annual intake with IMR, ID and MMR. Scatter plots were drawn to analyse the correlation between the medical school density of each province (log transformed) and its health care indicators- IMR, ID and MMR. The density of both ‘old medical schools’ as well as that of ‘new medical schools’) was used in creating scatter plots to separately observe the effect of both on provincial health profile.

#### District level analysis

Distribution of district level indicators was compared between districts with and without medical school till date. To study the pattern of expansion of medical schools in last decade districts were classified as group ‘A’ if they had any medical school in 2009 (or ‘old medical school’) and group ‘B’ if they did not have any medical school in 2009 (or ‘old medical school’). Proportion of districts which got a ‘new medical school’ during last decade (2009 to 2019) was compared between group A and B districts using Pearson Chi-square test.

To see the effect of new medical schools on district level indicators, distribution of district level population and household profile as well as health care indicators was compared between districts with and without medical school in 2019. To see the effect of new medical school distribution of district level indicators was compared separately for group ‘B’ districts with and without a new medical school in last decade (2009 to 2019). Non parametric approach (with application of Mann-Whitney U test) was used for comparison of district level indicators.

Software used: QGIS Desktop version 2.18.24, IBM SPSS Statistics version 25, Google Earth and Arc-Map version 10.

### Ethical approval

The present study uses information obtained from online open access databases available on the websites of the following: the Medical Council of India, National Institution for Transforming India (NITI), the 2011 Census, the National Family Health Survey - 4 (2015–16) and GADM database. The study was approved by the Institutional Ethics Committee of R.D.Gardi Medical College, Ujjain Madhya Pradesh, India.

## Results

### Spatio-temporal distribution and density of medical schools in India

**Fig. 1:**
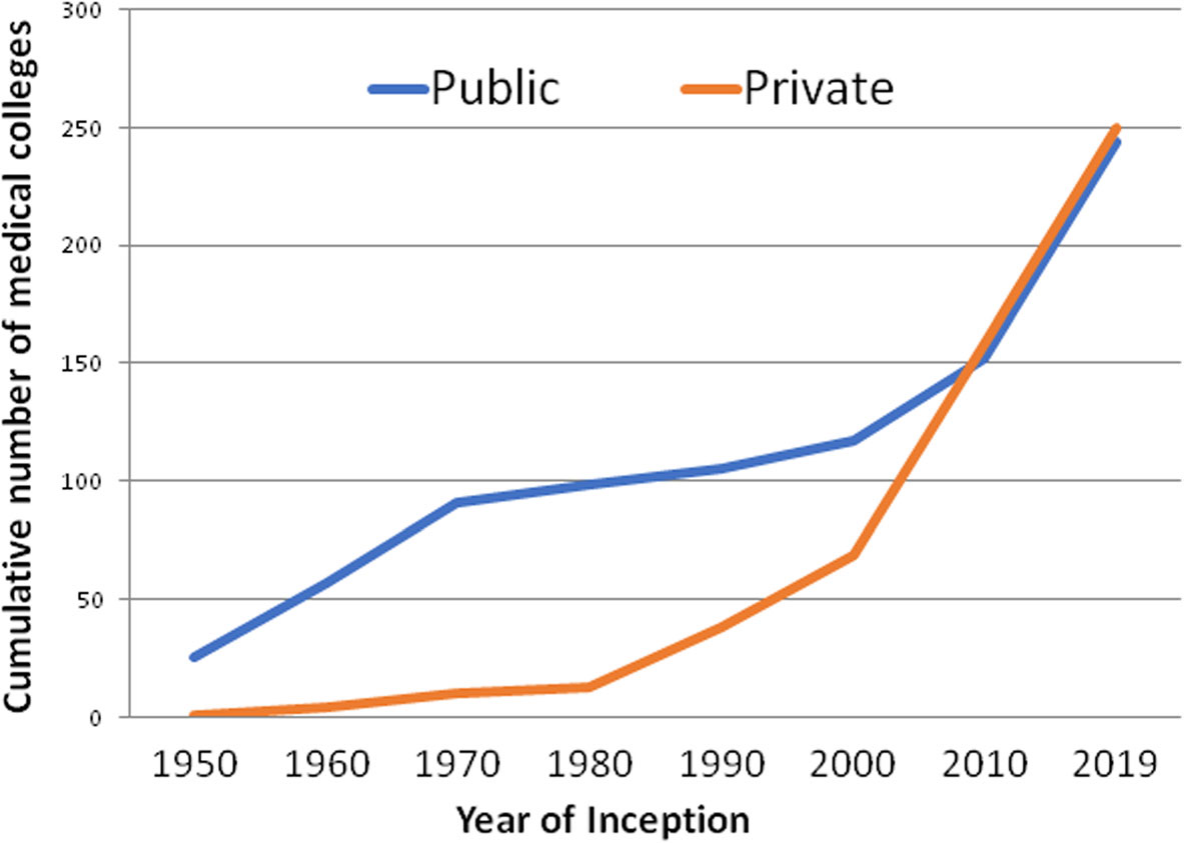
Distribution of medical schools in public and private sector as per year of inception

In 2019 there were 494 medical schools in India out of which 207 schools (41.98%) were opened in the last decade i.e. between 2009 till April 2019. Highest number of new medical schools i.e. 44 was established in 2016. The number of public sector schools was higher before 2009. But in 2010 the number of medical schools in public and private sector became almost similar and in the last decade the growth in their number has been parallel (Fig. 1).

A total of 63,250 students were enrolled for medical education in the academic year 2018–19. Annual intake was zero for forty (8.1%) medical schools while the number of students admitted into the remaining medical schools ranged from 50 to 250 (median 150). Overall annual intake per 10 million population was 522.35. New medical schools contributed 20,650 (32.65%) to the total number of enrolments done in academic year 2018–19.

**Fig. 2:**
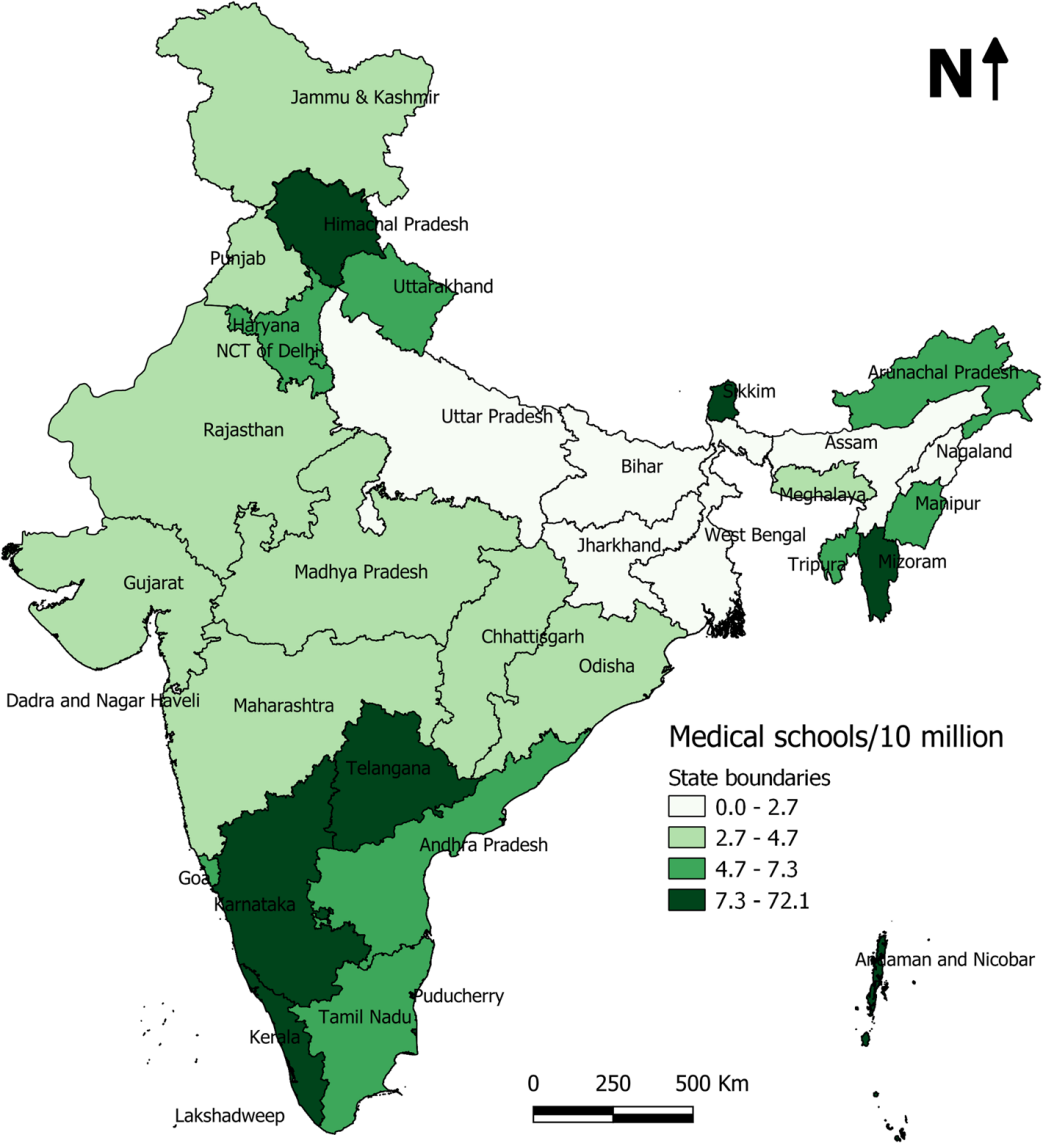
Province-wide distribution of medical school density in India

The 494 medical schools catered to a total population of 1,210,854,977 and accordingly the medical school density in India amounted to 4.08 per 10 million population. But medical schools were not evenly distributed in the country. Figure 2 shows the province-wide distribution of medical schools/ 10 million population in India. A geographically continuous belt of provinces with low medical school density can be seen in the North Eastern part of the country (Uttar Pradesh, Bihar, Jharkhand, West Bengal, Assam and Nagaland). It was evident from the data that no medical schools existed in the province of Nagaland and UTs of Dadra and Nagar Haveli, Daman and Diu, Lakshadweep. In other province/UTs the density of medical schools per 10 million population revealed a wide range from 0.92 (Jharkhand) to 72.12 (Puducherry). Of the total 36 provinces and Union territories in India, 16 had less medical school density than the national value of 4.08 per 10 million population.

**Fig. 3:**
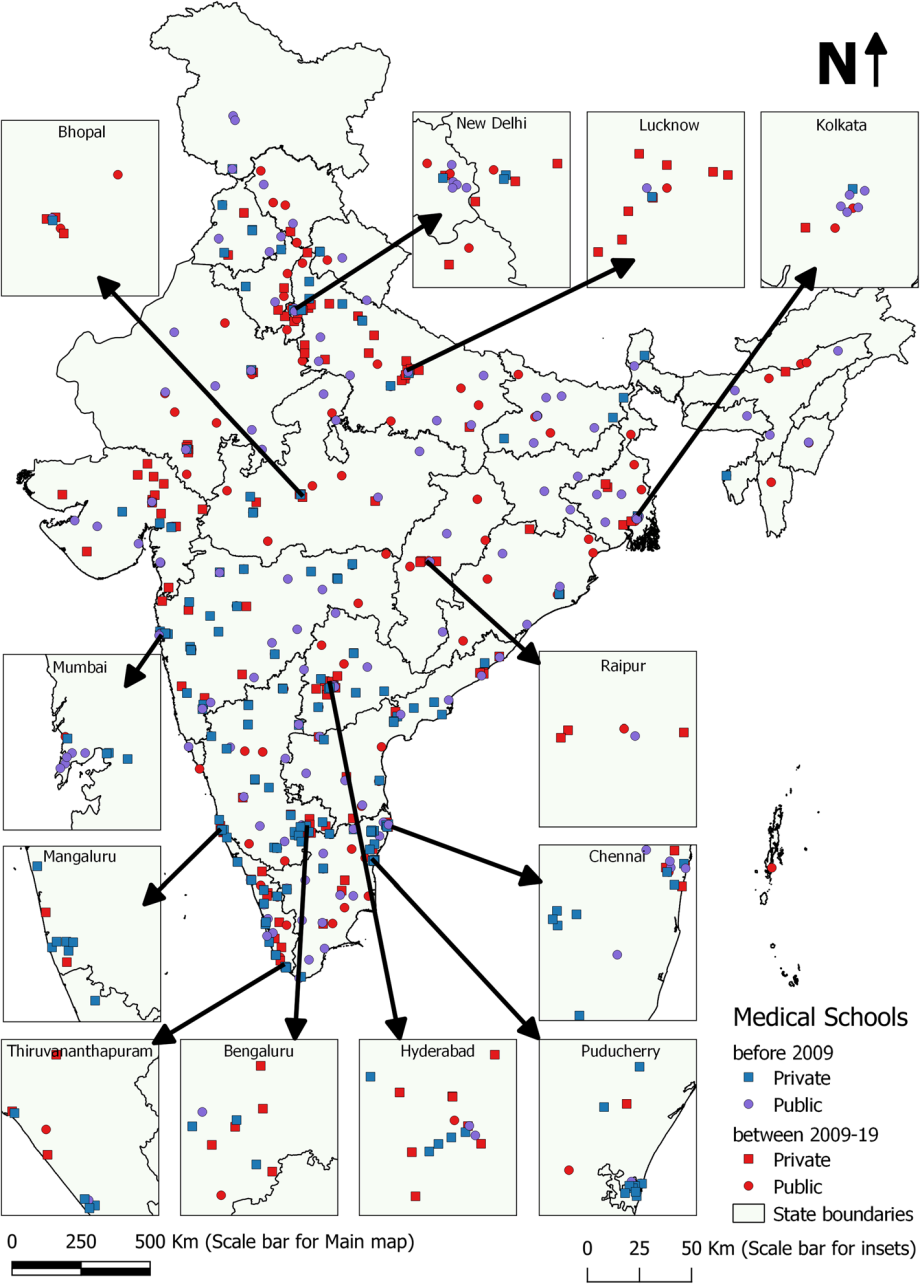
Geographic distribution of new and old medical schools in India- highlighting their spatial orientation in and around major cities and provincial capitals

Figure 3 shows the geographic distribution of old and new medical schools (in public and private sector) in India. It was revealed that medical schools were situated in clusters in certain parts of the country while large areas had no medical schools at all. This pattern was especially true for private medical education sector. 60.6% of all privately owned medical schools were located in the southern part of India including Maharashtra, Andhra Pradesh, Telengana, Karnataka, Kerala, Tamil Nadu and Puducherry. Results of Near neighbourhood analysis revealed significant clustering for both schools built before 2009 (Nearest neighbourhood ratio or NNR of 0.43 and Z score − 18.37) as well as for the schools opened between 2009 to 2019 (NNR 0.62 and Z score − 10.50).

### Comparison of the geographic distribution of new medical schools with that of old medical schools

#### Clustering in and around province capitals and major Indian cities

Figure 3 also reveals the clustering of medical schools in and around major cities and provincial capitals. In the insets the spatial distribution of medical schools in major metropolitan cities of India has been shown. For instance, the inset around Delhi shows that 9 new medical schools were built in the last decade even when there were already 8 existing medical schools established before 2009. Similar pattern were observed in the insets around other metropolitan cities like Chennai (5 new medical schools in addition to 7 existing ones), Hyderabad (8 new medical schools in addition to 7 existing ones), Bengaluru (6 new medical schools in addition to 4 existing ones), Mumbai (9 existing and 1 new medical school) and Kolkata (3 new medical schools in addition to 6 existing ones). Central and Northern provinces of Madhya Pradesh, Chhattisgarh and Uttar Pradesh (having poorer health and economic indicators) also revealed similar pattern at their respective provincial capitals (Bhopal, Raipur and Lucknow).

#### Clustering of new medical school in the vicinity of old medical schools

Distance matrix revealed that the median distance of a new medical school from its nearest old medical school was just 22.81Km with an IQR of 6.29 to 56.86Km. Out of 207 new medical schools 139 (67.15%) were located within 50Km of an old medical school. This pattern was more marked for private medical schools (Median 19.4, IQR 7 to 38 Km). This implied that medical schools are being built in clusters around already established medical schools.

### Effect of density and annual intake of medical schools on province wise health indicators

Tables 2 and 3 show association of the province/UT level medical school density per 10 million population and the total annual intake of medical schools per 10 million with health indicators viz. IMR, ID and MMR. Comparison done separately for old medical schools and new medical schools indicates that correlation is highly significant for old medical schools statistics.

**Table 2:** Correlation between medical school density and MMR, ID and IMR

Medical schools per 10 million population	Spearman Correlation	MMR	ID	IMR
Total	Correlation Coefficient	−.623^a^	.384^b^	−.365^b^
	Sig. (2-tailed)	0.01	0.02	0.03
New (2009–19)	Correlation Coefficient	−0.42	0.13	0.09
	Sig. (2-tailed)	0.09	0.45	0.61
Old (before 2009)	Correlation Coefficient	−.570^b^	.444^a^	−.329^b^
	Sig. (2-tailed)	0.01	0.01	0.05

*MMR* Maternal Mortality ratio, *ID* Institutional deliveries, *IMR* Infant mortality rate

Spearman’s rho

^a^. Correlation is significant at the 0.01 level (2-tailed)

^b^. Correlation is significant at the 0.05 level (2-tailed)

**Table 3:** Correlation between annual intake of medical schools with MMR, ID and IMR

Annual intake per 10 million population	Spearman Correlation	MMR	ID	IMR
Total	Correlation Coefficient	−.637^a^	.547^a^	−.390^b^
	Sig. (2-tailed)	0.00	0.00	0.02
New (2009–19)	Correlation Coefficient	−0.38	0.18	0.09
	Sig. (2-tailed)	0.12	0.30	0.59
Old (before 2009)	Correlation Coefficient	−.640^a^	.518^a^	−.342^b^
	Sig. (2-tailed)	0.00	0.00	0.04

*MMR* Maternal Mortality ratio, *ID* Institutional deliveries, *IMR* Infant mortality rate

Spearman’s rho

^a^. Correlation is significant at the 0.01 level (2-tailed)

^b^. Correlation is significant at the 0.05 level (2-tailed)

**Fig. 4:**
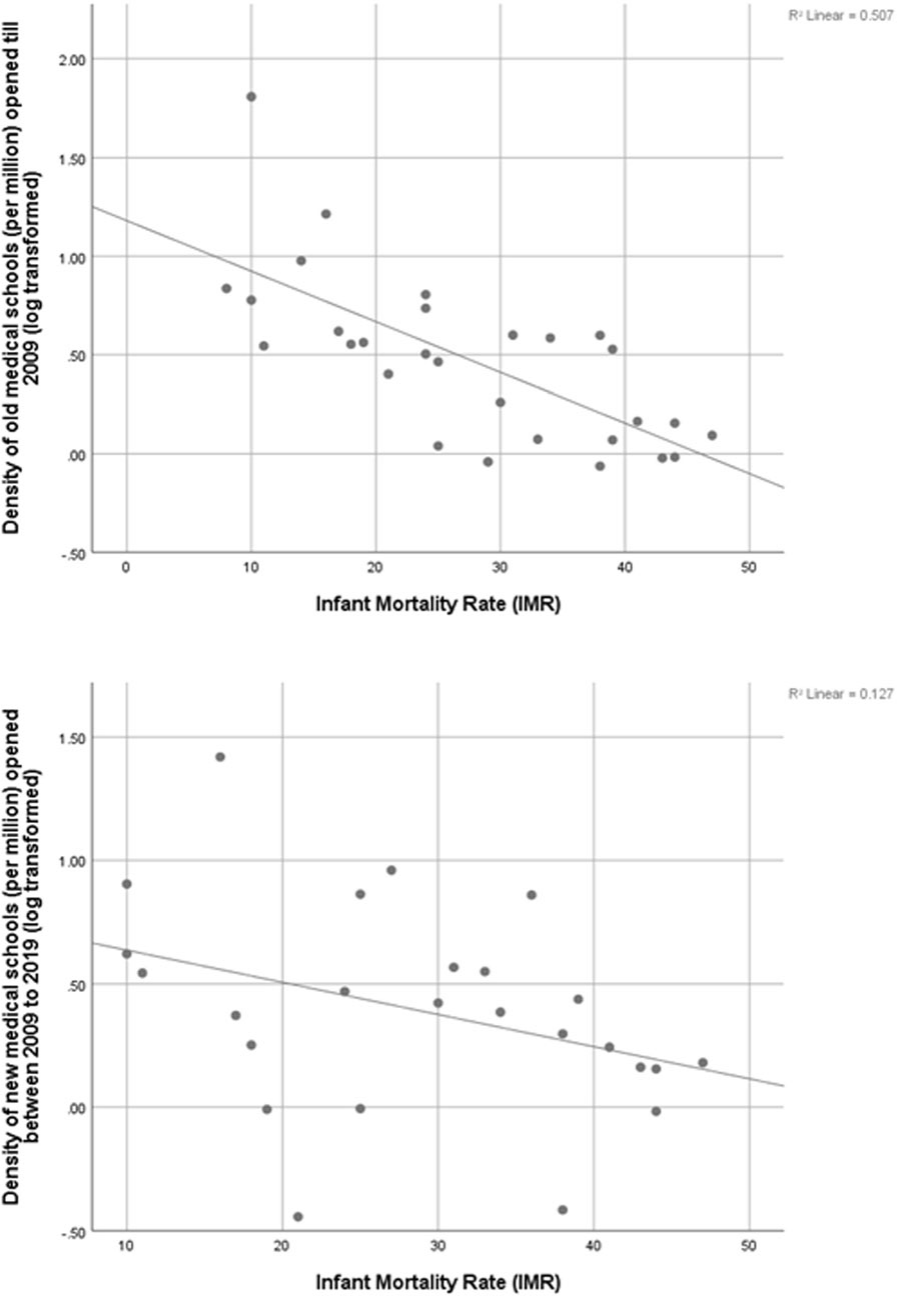
Medical school density of provinces and their Infant Mortality Rate (IMR)

**Fig. 5:**
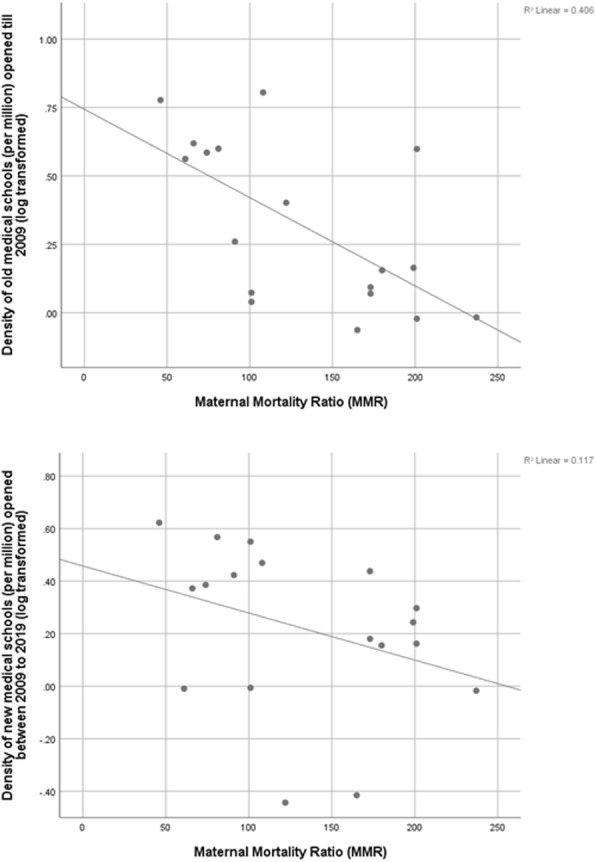
Medical school density of provinces and their Maternal Mortality Ratio (MMR)

**Fig. 6:**
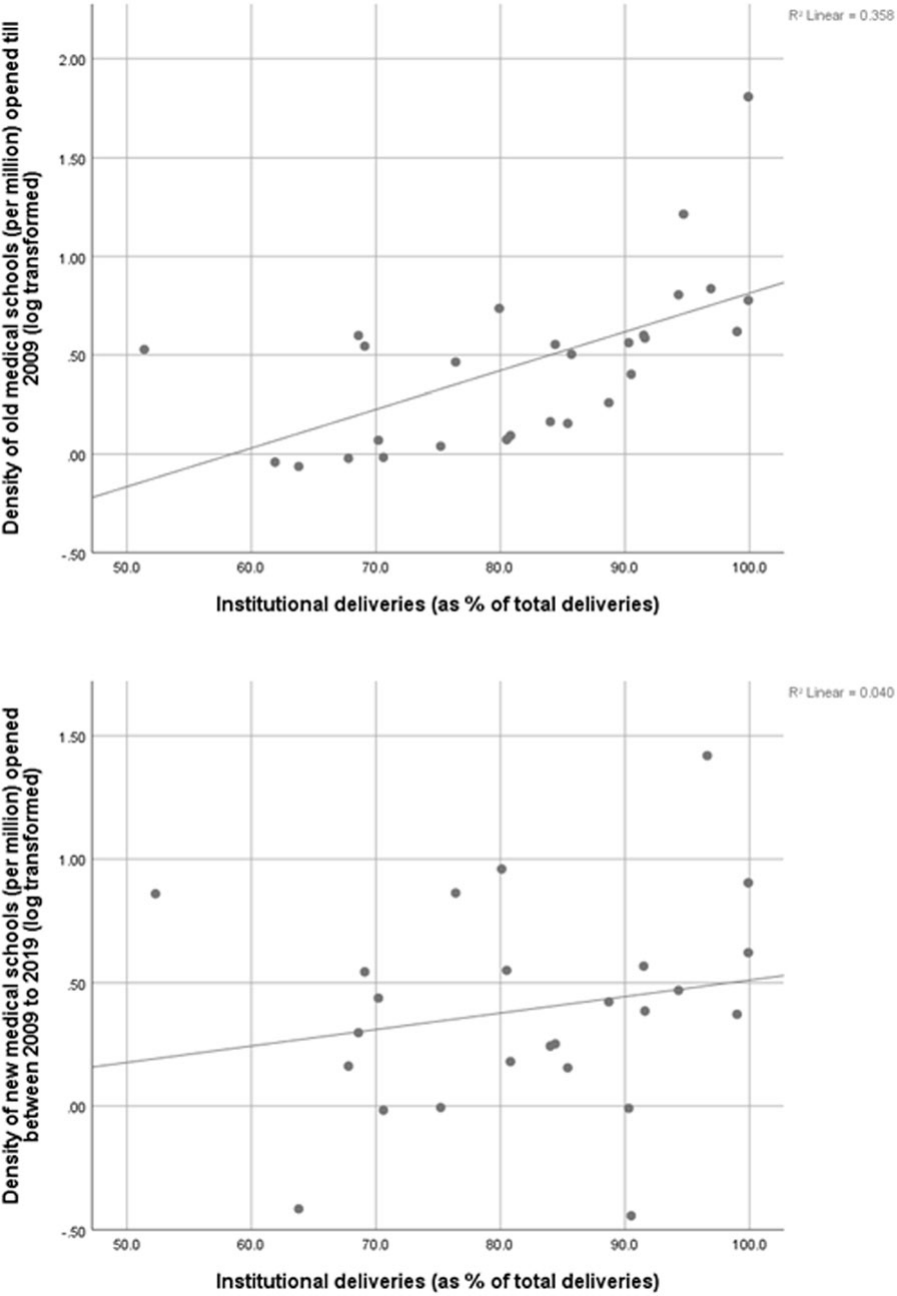
Medical school density of provinces and the percentage of Institutional Delivery (ID)

Figures 4, 5 and 6 shows the scatter diagram of medical school density (per million) (log transformed) of the provinces and respective province level health indicators viz. IMR, ID and MMR. Linear (positive) correlation as indicated by R^2^ was observed between percentage of institutional deliveries of the province and its medical school density and the strength of relationship is higher in case of medical schools established prior to 2009. Similarly negative correlation can be observed between MMR and IMR on one hand and log transformed medical school density on the other hand indicating that maternal and infant mortality rates are reduced when public has higher access to medical schools.

### Comparison of socioeconomic and health care profile of districts with and without medical schools

Table 4 shows the breakdown of districts according to availability of medical school before and after 2009. Among the total 640 districts in India only 177 (27.6%) had at least one medical school before 2009 within their geographic boundaries (group A). Of the rest 463 districts (group B) which did not have a medical school till 2009, medical schools were opened in only 93 (20.1%) districts leaving behind 370 (79.9%) districts which as yet did not have a single medical school. The proportion of districts which did not get a new medical school was significantly higher among the districts without medical school before 2009 (group B, 79.9%) as compared (using Pearson Chi-square test) to their counterparts (group A, 65%) (*p* value < 0.001).

**Table 4:** The distribution of districts according to availability of medical school before and after 2009

New Medical School	Old Medical School	
	Yes (Group A)	No (Group B)
	No (%)	No (%)
Yes	62 (35.0)	93 (20.1)
No	115 (65.0)	370 (79.9)*
Total	177	463

*Pearson Chi-square test *p* value < 0.001

Table 5 shows the distribution of population and household profile among districts with and without medical school in 2019. It reveals that the median value of all the population and household profile indicators were significantly better in districts where medical schools were opened. Table 6 shows distribution of population and household profile indicators among districts which did not have a medical school at the beginning of last decade i.e. in 2009 (group B). The distribution is presented for group B districts with and without a new medical school. Again the median value of all the population indicators was significantly better in districts where new medical schools were opened.

**Table 5:** Distribution of population and household profile indicators among districts with and without medical school in 2019

Characteristics All districts	District with at least one medical school till 2019				Districts which did not have a medical school till 2019			
(***N*** = 640)	N	Median	IQR		N	Median	IQR	
Households with electricity	270	96.9^a^	91.5	98.9	370	90.4	77.0	97.8
Households using improved sanitation facility	270	52.1^a^	38.6	68.6	370	38.9	23.3	62.1
Households using clean fuel for cooking	270	46.6^a^	29.0	63.3	370	22.9	15.5	40.5
Women who are literate	270	72.6^a^	63.1	82.0	370	66.5	54.8	77.1

^a^Very highly significant, significant Mann-Whitney U test

**Table 6:** Distribution of population and household profile indicators among group B districts (which did not have a medical school in 2009) with and without a new medical school

Characteristics Districts which did not have a medical school in 2009 (***N*** = 463)	Opened a new medical school during 2009–2019				Districts which did not have a medical school till date			
	N	Median	IQR		N	Median	IQR	
Households with electricity	93	95.2^b^	85.8	98.7	370	90.4	77.0	97.8
Households using improved sanitation facility	93	48.8^b^	32.9	68.2	370	38.9	23.3	62.1
Households using clean fuel for cooking	93	32.5^a^	21.2	48.4	370	22.9	15.5	40.5
Women who are literate	93	69.8^b^	58.6	78.7	370	66.5	54.8	77.1

^a^Very highly significant,^b^significant Mann-Whitney U test

Table 7 compares the distribution of public health indicators among districts with and without medical school in 2019. It was observed that districts with medical schools showed better performance in all health indicators as compared with districts with no medical school. Table 8 compares the distribution of public health indicators among districts which did not have a medical school at the beginning of last decade i.e. in 2009 (group B). The findings suggested that establishment of new medical schools also had a positive impact on health outcome of the district. However performance of only some health indicators was significantly affected.

**Table 7:** Distribution of public health indicators among districts with and without medical school in 2019

Characteristics All districts	District with at least one medical school till 2019				Districts which did not have a medical school till 2019			
(N = 640)	N	Median	IQR		N	Median	IQR	
Mothers who had full antenatal care	269	27.5^a^	11.9	39.2	369	12.6	5.3	26.8
Mothers who received postnatal care from a doctor/nurse/LHV/ANM/midwife/other health personnel within 2 days of delivery	269	68.8^a^	58.1	78.8	370	58.4	45.1	72.2
Children who received a health check after birth from a doctor/nurse/LHV/ANM/ midwife/other health personnel within 2 days of birth	269	24.3^a^	16.8	33.2	370	20.1	11.4	31.4
Births assisted by a doctor/nurse/LHV/ANM/other health personnel	269	89.2^a^	79.6	95.6	370	79.8	67.6	90.9
Children with fever or symptoms of ARI in the last 2 weeks preceding the survey taken to a health facility	185	76.9^a^	68.2	84.1	299	71.2	60.0	80.8
Women Age 15–49 Years Who Have Ever Undergone Examinations of Cervix	268	22.6^a^	13.1	34.1	369	15.5	9.4	26.5
Women Age 15–49 Years Who Have Ever Undergone Examinations of Breast	268	8.1^a^	4.4	15.8	368	6.0	3.5	10.5

^a^Very highly significant

**Table 8:** Distribution of public health indicators among group B districts (which did not have a medical school in 2009) with and without a new medical school

Characteristics Districts which did not have a medical school in 2009 (N = 463)	Opened a new medical school during 2009–2019				Districts which did not have a medical school till 2019			
	N	Median	IQR		N	Median	IQR	
Mothers who had full antenatal care	92	18.8^a^	7.6	34.3	369	12.6	5.3	26.8
Mothers who received postnatal care from a doctor/nurse/LHV/ANM/midwife/other health personnel within 2 days of delivery	92	65.0^a^	55.5	75.0	370	58.4	45.1	72.2
Children who received a health check after birth from a doctor/nurse/LHV/ANM/ midwife/other health personnel within 2 days of birth	92	22.2	15.8	31.3	370	20.1	11.4	31.4
Births assisted by a doctor/nurse/LHV/ANM/other health personnel	92	84.6^a^	75.6	92.3	370	79.8	67.6	90.9
Children with fever or symptoms of ARI in the last 2 weeks preceding the survey taken to a health facility	72	76.0^a^	66.6	82.0	299	71.2	60.0	80.8
Women Age 15–49 Years Who Have Ever Undergone Examinations of Cervix	91	22.6^a^	12.9	28.9	369	15.5	9.4	26.5
Women Age 15–49 Years Who Have Ever Undergone Examinations of Breast	91	8.7^a^	4.4	15.6	368	6.0	3.5	10.5

^a^significant Mann-Whitney U test

## Discussion

Findings from our study indicate that uneven distribution of medical schools is prominent in India and that the presence of a medical school can have a positive effect on the health profile of the surrounding community. In the following sections the main insights generated during analysis are discussed.

### Concentration of medical schools in southern provinces of India

Findings of this study reaffirm the conclusions drawn by previous studies regarding the concentration of medical schools in southern provinces of India. In 2010 Ananthakrishnan revealed that only 11% of all medical schools were located in northern and eastern provinces whereas 61% were clustered in the southern provinces [17]. Another study published in 2014 showed that the corresponding proportions in the aforementioned northern and southern provinces were 11.8 and 56.3% respectively [19]. We found that the six southern provinces of Maharashtra, Andhra Pradesh, Telengana, Karnataka, Kerala and Tamil Nadu together with the Union Territory of Puducherry account for 52% of all medical schools in India even though only 30% of the country’s population resides in these areas. Although a positive trend can be noted in the afore-mentioned data in form of decreasing proportion of medical schools clustered in south India, the change over the last decade has not been substantial.

This disparity in medical school distribution has been attributed by previous studies to the differences in regional political will and socioeconomic prosperity [26, 33]. While that might be true, the abundance of medical aspirants noticed in these regions might also contribute to the skewed distribution of medical schools. As per statistics published by the Press Information Bureau, in 2019 almost 50% of candidates who registered for the National Eligibility Cum Entrance Test, a mandatory qualification exam for admission into undergraduate medical degree programme (MBBS) in any medical school of India, belonged to the above mentioned seven provinces and union territories [34]. Our findings also prove that more than 60% of all private medical schools in India are located in the afore-mentioned southern provinces and of all the medical schools in these provinces majority (61%) belong to the private sector. It might be because investors of private medical schools are not only motivated by the paying capacity of the community where the proposed medical school will be established but also by the availability of sufficient number of prospective students. Hence the higher medical school density observed in these areas might be a reflection of the attempt by private investors to capture the bulk of medical aspirants in India.

Even though we have used medical school density as the independent variable to analyse distribution of medical schools in India, density alone might not be sufficient to ascertain need for medical school in an area as is seen in north-eastern India. Of the eight north-eastern provinces namely Arunachal Pradesh, Assam, Manipur, Mizoram, Meghalaya, Nagaland, Sikkim and Tripura; only Nagaland and Assam have medical school density in the lowest quintile. Sikkim and Mizoram have medical school density well above the national average. Densities of these two provinces are high despite their having only one medical school each because of their small population base compared to other Indian provinces. However because of the hilly terrain prevalent in these regions providing medical access to the sparsely populated, remote, far flung areas is a challenge and has been considered one of the major reasons, besides shortage of health professionals, of poor health performance of the north-eastern provinces [35].

### Clustering of medical schools in and around major cities and provincial capitals

Mapping of medical schools in India in this study revealed that medical education is concentrated in major cities and provincial capitals of the country. Researchers have previously studied the differential distribution of medical schools in urban and rural areas [20]. To extend their work, we have analysed the medical school density of metropolitan cities of India. The findings paint a dismal picture for health care in India. The clustering of medical schools in cities adversely affects the distribution of health workforce in the country. A recent study showed that prohibited distance from qualified HRH and consequent expenditure needed for transport are the key contributors to the popularity of unqualified health care workers [36]. Chen LC pointed out that severe mal-distribution can harm not only the disadvantaged, but also high-income populations. Over-abundance of specialized health professionals in cities can lead to unnecessary diagnostic procedures, over-prescription of drugs and even iatrogenic diseases plaguing the rich and poor alike while residents of less urbanised areas suffer from lack of adequate health resource availability [37]**.**

High medical school density in cities can be due to the easy availability of qualified health staff required for establishment of a new medical school according to pre-requisite guidelines of MCI [38]. Previous studies have attempted to identify the reasons behind preferential selection of urban practice locality among health professionals in India [39]. Reported barriers to rural practice include poor pay, professional isolation, limited opportunities of career growth for self and family and lack of urban amenities. Diwan et al. in 2013 showed that more than half of the medical students wish to set up practice away from rural areas [40]**.** However it has been concluded by numerous studies that alumni of medical schools located in rural regions are better inclined for rural practice [41]**.** Hence WHO recommends establishing new medical schools away from major cities and capitals [42]. This guideline is especially applicable to India considering the dearth of medical professionals in rural areas [14].

### Clustering of new medical schools in the vicinity of established medical schools

As shown by results of distance matrix analysis, new medical schools opened in the last decade (2009–2019) in India have been concentrated with 50Km radius of already existing medical schools. This trend is more prominent among the privately owned new medical schools. One reason for this phenomenon might be the availability of better infrastructure in the locality which attracted opening of the already established schools. Also if the existing medical schools are functioning well then it can be argued that a new medical school might also have a higher chance of success and such a guarantee is bound to lure commercially oriented investors in the absence of any prohibiting regulation**.** Furthermore it has been reported by previous studies that private medical schools in India have the tendency to lure teaching faculty from existing medical schools or even just utilise their names to avoid being de-recognised by the MCI during inspection of their amenities [26, 43]. Hence a dense clustering of newly opened private medical schools is commonly seen. However as pointed out by the Flexner report which provides benchmark guidelines for the planning of medical education sector increasing number of medical schools in a limited geographic area leads to undesirable competition for faculty while providing no additional benefits and hence is but a mere wastage of precious health care resources [44].

### Positive effect of medical school on health care

Provinces with higher medical school density show better performance in major health indicators like maternal mortality ratio (MMR), Infant mortality rate (IMR) and percentage of Institutional delivery. Similar pattern can be observed in districts with medical schools. This can be because medical schools in India are associated with teaching hospitals which deliver tertiary care services. Medical schools also complement local primary health care by providing outreach services in the defined geographic area where they are located [19]. Hence morbidity and mortality statistics of the community decrease due to timely availability of requisite medical care for time-sensitive cases like caesarean sections for pregnant women, trauma [45] and other medical emergencies like Acute Myocardial infarction [46]. Studies have also shown the beneficial impact of access to a medical school on prognosis of other diseases like Breast cancer [47].

The role played by medical schools in improving health of the local community gets more pronounced as the duration for which the medical school has been functioning increases. This can be explained by the fact that medical schools in India are allowed to start with limited infrastructure as well as limited annual intake of students [21]. Later on based on their performance which is evaluated by MCI during routine inspections they are allowed to expand [21]. Hence it can be expected that a medical school in India will take some time to reach its optimal potential which is seen in the differential health impact of new versus already established medical schools.

It is evident from the above discussion that reformation of the rules for establishment of new medical schools in India is critical. The reformed rules should promote opening of new medical school in those districts which still do not have any as opposed to establishment of surplus new schools in districts with existing old schools. Similarly opening of new schools should be preferentially allowed in districts in the Northern and North-eastern parts of India where the medical school density is much lower compared to the southern areas. However, according to available MCI regulations a unitary campus of 20 acres is required for grant of permission to open a new medical school [21]. Relaxation is allowed in north-eastern provinces and metropolitan cities [21]. Although allowing concessions for the north-eastern region is an appropriate move, extending the same courtesy to metropolitan cities that already have medical school will only promote further clustering of medical schools in areas which already are plagued by over-abundance of medical professionals.

The use of location allocation analysis models [2] can be beneficial in delineating areas which most require the establishment of new medical schools and accordingly grant ‘essentiality certificate’. Such decisions are currently being taken by the national and provincial governments of India based on political or pragmatic considerations. However studies have shown that bias is common in such decisions making them far from optimal [48, 49]. This is also supported by our finding that Population and household profile indicators were significantly better in districts where medical schools were opened. Although mathematical modelling for prioritising areas for preferential allotment of limited resources has been traditionally considered too sophisticated for use in developing countries like India, recent studies have proven their efficacy in the locational decision-making process. Hence integration of such methods into the regulations guiding establishment of medical schools in India should be considered.

However it should be remembered that there are a multitude of other factors which affect the health profile of a community and thus in turn influence the requirement of a new medical school in the locality. Since in our study we have only analysed existing data, the complex interaction between health care facilities including medical school and the diverse spectrum of other determinants of health such as the overall socio economic profile of the district, education levels of the native community, environmental and genetic factors could not be elucidated. Hence concluding that establishment of a medical school alone will be sufficient to significantly improve the public health status of a community is impractical.

Similarly another limitation of the current study is that the role played by other members of the health workforce has not been taken into consideration. Analysis of the complex relationship between medical school density and community health profile is incomplete without understanding factors like physician migration after study completion which determine the ultimate size and composition of the health workforce in any given area. Future studies should attempt to obtain more comprehensive information about the correlation between annual intake and serving physician output in order to determine optimal medical school density. Furthermore, the mere presence of a medical school and hence trained medical personnel to dispense health care in a particular area does not necessarily translate into a significant betterment in the quality of health care delivery. Published literature has revealed that without proper planning and time allocation, clinical teachers in medical schools struggle to balance their dual responsibilities of patient care and medical education, thus limiting their impact on local health care delivery [50]. Similarly the physician capacity of medical students trained through the traditional medical education system has been widely debated both globally and in India [51–55]. Armed only with theoretical conceptual knowledge, medical school pass outs often lack procedural knowledge which forms the very basis of clinical case solving [56]. Even with the advent of competency based curriculum, the undue focus on scientific knowledge of trainee physicians has crippled their skills in meeting the social, ethical and humanistic aspects of healthcare needs [57, 58]. Another important aspect of medical education that can significantly affect the quality of healthcare delivery is the judicious selection of potential students [59] which is already questionable in India [54]. It is quite possible that establishing further medical schools without quality control especially in the selection process of candidates will merely result in unworthy candidates gaining entrance and thus adversely affect health care. Hence simply opening new medical schools, without paying sufficient attention to maintenance of quality of both student selection and thereafter education, might not be a panacea to the healthcare woes of the Indian population.

## Conclusion

In this study we have mapped the locations of medical schools using geographic information system (GIS) to elaborate the spatial distribution of Indian medical education system and its influence on public health. Findings reveal that wide geographic areas in the Northern and North Eastern part of the country either have low medical school density or are completely devoid of medical schools. In contrast, medical schools are clustered in the southern provinces of the country. Within each province medical schools are again concentrated in and around major cities and capital regions. Districts with poorer population and household indicators have fewer medical schools. The shortage of trained health workforce in smaller cities, semi-urban and rural areas can be partly attributed to this pattern of aggregation. This mal-distribution is further compounded by selective opening of new medical schools within the catchment area of already established medical schools.

Keeping in mind the strengths and limitations of this study, it can be concluded that medical schools might have a positive influence on public health. Equitable regulation of grant of permission to open future medical schools might therefore be important to solve the health manpower crisis in India and thus lead to achievement of universal health coverage and finally better health. It is therefore recommended that further research be conducted to comprehensively understand the entire scenario. Based on insights thus generated the rules guiding medical school establishment in India can be finalised and included in the future strategies.

## Data Availability

The present study uses information obtained from online open access databases available on the websites of the following: The Medical Council of India, National Institution for Transforming India (NITI), the 2011 Census, the National Family Health Survey - 4 (2015–16) and GADM database. The same can be retrieved by interested researchers.

## Abbreviations

UHC: Universal Health Coverage
HRH: Human Resource for Health
MCI: Medical Council of India
ID: Institutional Delivery
NITI: National Institution for Transforming India
NFHS: National Family Health Survey
MMR: Maternal Mortality Ratio
IMR: Infant Mortality Rate
UT: Union Territories
